# Supplementary material for: MRI- and histologically derived neuroanatomical atlas of the Ambystoma mexicanum (axolotl)
Source: Sci Rep. 2021 May 10;11:9850. doi: 10.1038/s41598-021-89357-3 (PMC8110773; doi:10.1038/s41598-021-89357-3)
Supplement: Supplementary file 1 — Supplementary Information. [file 41598_2021_89357_MOESM1_ESM.pdf]

Supplementary information

MRI- and histologically derived neuroanatomical atlas of the *Ambystoma mexicanum* (axolotl)

Iván Lazcano[1]*, Abraham Cisneros-Mejorado[1 2], Luis Concha[1], Juan José Ortíz Retana[1], Eduardo A. Garza-Villarreal[1]*, Aurea Orozco[1]

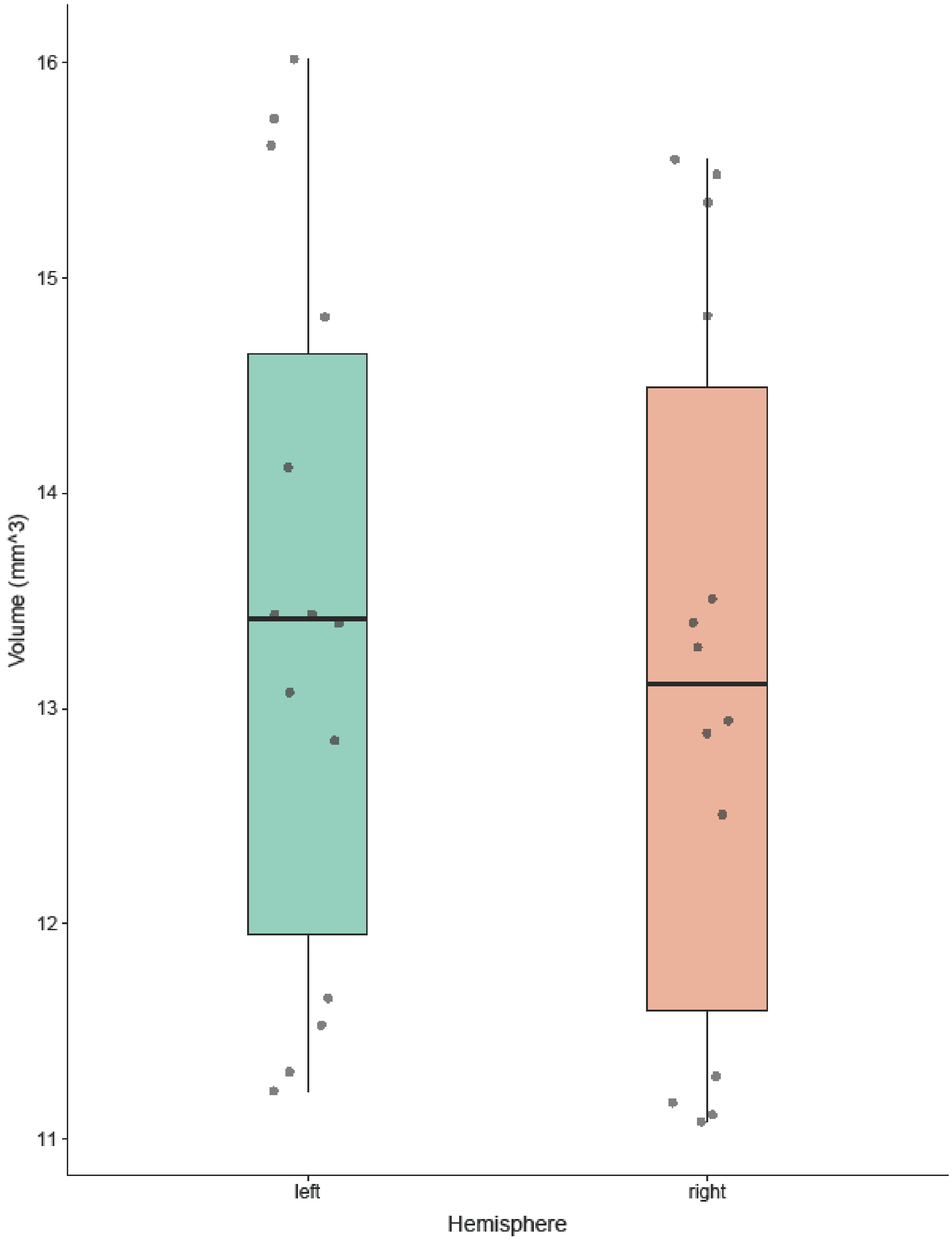

Supplementary Figure S1. Average volume (mm^3) of left and right hemispheres from 14 juvenile axolotls.

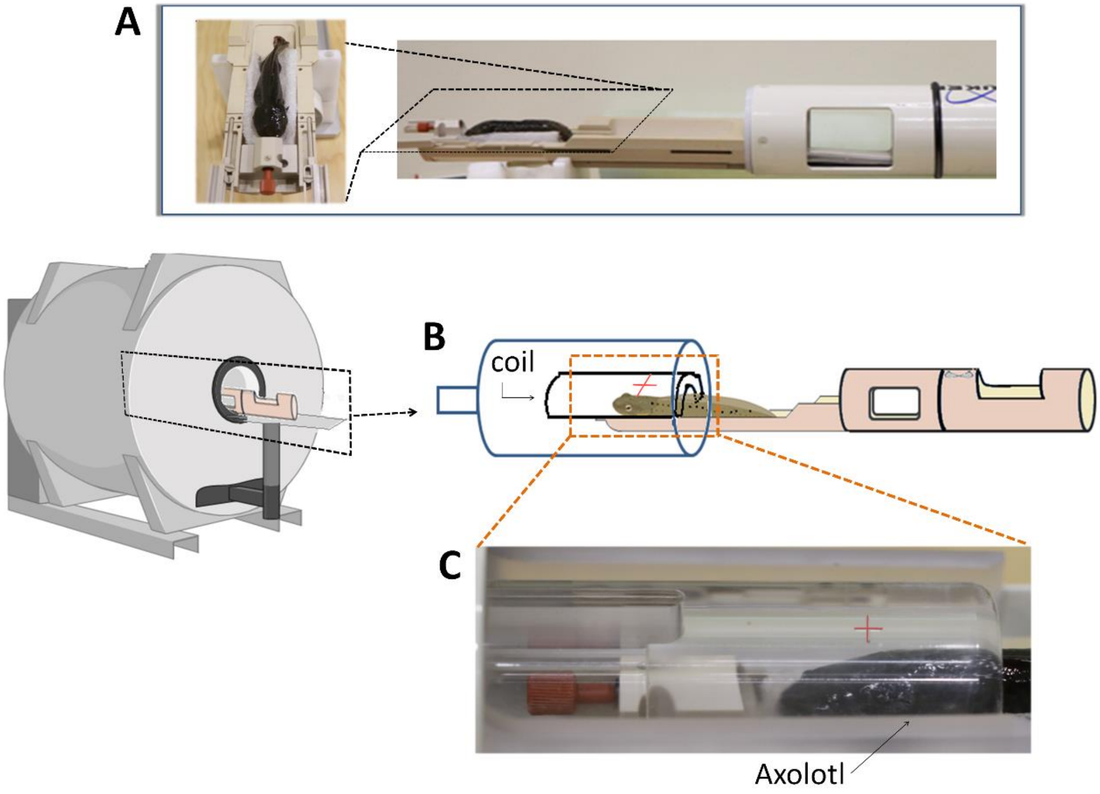

Supplementary Figure S2. Once anesthetized, the axolotl is placed on a plastic stretcher over a holder, in (A) the axolotl is observed in a top view (left) and from a lateral view (right). Then, it is inserted into the scanner, in such a way that the center of coil (Helium-cooled two-channel rat-head coil: Bruker Cryopobe) is approximately 1 cm from the eyes towards the caudal part (B). In (C) the placement of the axolotl in the coil-simulator is shown, the red mark indicates the center of the coil.

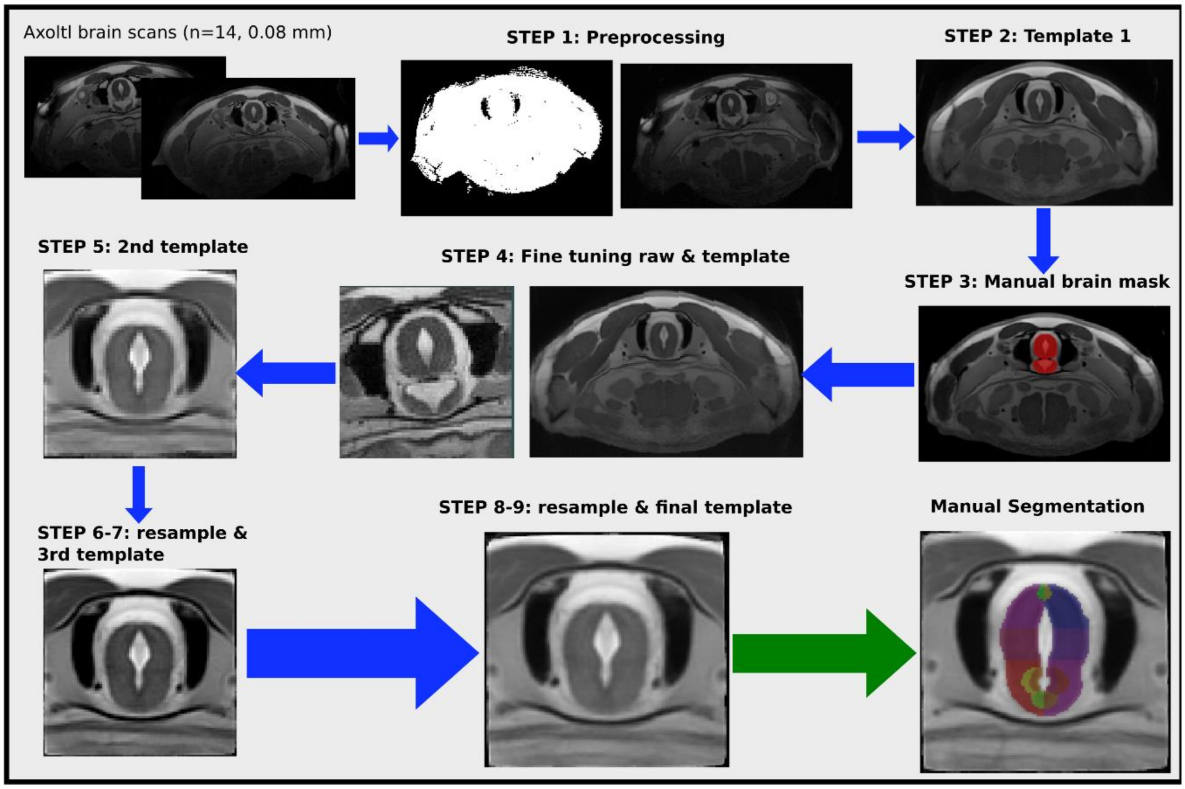

Supplementary Figure S3. Pipeline for template construction. Flow chart of the process used to construct the template used for segmentation. Details about the steps in the Methods Section.

Supplementary Table 1

List of ROIs, abbreviations, references, hemisphere, mayor structure, volume, standar deviation (SD) and color

Structure	***Abbreviation***	***Hemisphere***	***Mayor structure***	***mean volume (mm^3)***	***SD***	***Color***
**olfactory nerve**	on (1)	Left	Olfactory bulb	0.161	0.028	
**glomerular layer**	g (1)	Left	Olfactory bulb	0.472	0.077	
**mitral cell layer**	m (1)	Left	Olfactory bulb	0.24	0.031	
**granule cell layer**	gc (1)	Left	Olfactory bulb	0.116	0.018	
**anterior olfactory nucleus**	aon (1)	Left	Olfactory bulb	0.139	0.016	
**pallium**	p (2)	Left	Telencephalon	3.286	0.0498	
**subpallium**	sp (sp)	Left	Telencephalon	1.179	0.149	
**medial part of amygdala**	amc (5,6)	Left	Telencephalon	0.068	0.009	
**pallial commissure**	cpa (6)	Left	Telencephalon	0.02	0.004	
**anterior commisure**	ca (6)	Left	Telencephalon	0.025	0.003	
**antertior preoptic nucleus**	npa (6,7)	Left	Telencephalon	0.101	0.01	
**lateral/medial forebrain bundle**	lfb (7)	Left	Telencephalon	0.702	0.083	
**choroid plexus**	cp (8)	Left	Telencephalon	0.171	0.034	
**thalamic eminence**	em th(6)	Left	Telencephalon	0.025	0.005	
**posterior preoptic nucleus**	ppn (7)	Left	Diencephalon	0.068	0.009	
**habenula**	hab (4,7)	Left	Diencephalon	0.041	0.005	
**thalamus**	th (4,7)	Left	Diencephalon	0.987	0.102	
**hypothalamus dorsalis**	hyth d (4,7)	Left	Diencephalon	0.872	0.105	
**paraventricular organ**	pvo (4,7)	Left	Diencephalon	0.052	0.008	
**pars dorsalis hypothalami**	pdh (4,7)	Left	Diencephalon	0.025	0.004	
**pars ventralis hypothalami**	pvh (4,7)	Left	Diencephalon	0.025	0.002	
**subcommissural organ**	so (4,7)	Left	Diencephalon	0.019	0.003	
**ependymal cell layer**	ecl (7)	Left	Diencephalon	0.254	0.232	
**optic chiasm**	och (7)	Left	Mesencephalon	0.023	0.003	
**tectum**	to (4,7)	Left	Mesencephalon	1.255	0.17	
**nucleus interpeduncularis**	nip (4)	Left	Mesencephalon	0.005	0.002	
**hypothalamus ventralis**	hyt v (4)	Left	Mesencephalon	0.193	0.028	
**pituitary**	hy (9,10)	Left	Endocrine	0.086	0.02	
**rhombencephalon**	rh(9)	Left	Rhombencephalon	0.516	0.058	
**nervous trigeminus**	V (4)	Left	Rhombencephalon	0.078	0.016	
**nervous lateralis anterior/nervous octavus**	VIII (4)	Left	Rhombencephalon	0.171	0.028	
**gray matter of medulla oblongata**	gmob (9)	Left	Rhombencephalon	0.685	0.08	
**withe matter of medulla oblongata**	wmob (9)	Left	Rhombencephalon	1.403	0.155	
**olfactory nerve**	on (1)	Right	Olfactory bulb	0.193	0.032	
**glomerular layer**	g (1)	Right	Olfactory bulb	0.419	0.068	
**mitral cell layer**	m (1)	Right	Olfactory bulb	0.24	0.039	
**granule cell layer**	gc (1)	Right	Olfactory bulb	0.119	0.017	
**anterior olfactory nucleus**	aon (1)	Right	Olfactory bulb	0.142	0.016	
**pallium**	p (2)	Right	Telencephalon	3.205	0.496	
**subpallium**	sp (2)	Right	Telencephalon	1.139	0.135	
**medial part of amygdala**	amc (5,6)	Right	Telencephalon	0.07	0.013	
**pallial commissure**	cpa (6)	Right	Telencephalon	0.023	0.003	
**anterior commisure**	ca (6)	Right	Telencephalon	0.027	0.005	
**antertior preoptic nucleus**	npa (6,7)	Right	Telencephalon	0.108	0.015	
**lateral/medial forebrain bundle**	lfb (7)	Right	Telencephalon	0.72	0.093	
**choroid plexus**	cp (8)	Right	Telencephalon	0.142	0.033	
**thalamic eminence**	em th(6)	Right	Telencephalon	0.024	0.004	
**posterior preoptic nucleus**	ppn (7)	Right	Diencephalon	0.07	0.008	
**habenula**	hab (4,7)	Right	Diencephalon	0.04	0.005	
**thalamus**	th (4,7)	Right	Diencephalon	1.006	0.135	
**hypothalamus dorsalis**	hyth d (4,7)	Right	Diencephalon	0.861	0.094	
**paraventricular organ**	pvo (4,7)	Right	Diencephalon	0.052	0.008	
**pars dorsalis hypothalami**	pdh (4,7)	Right	Diencephalon	0.022	0.004	
**pars ventralis hypothalami**	pvh (4,7)	Right	Diencephalon	0.026	0.002	
**subcommissural organ**	so (4,7)	Right	Diencephalon	0.019	0.003	
**ependymal cell layer**	ecl (7)	Right	Diencephalon	0.26	0.031	
**optic chiasm**	och (7)	Right	Mesencephalon	0.021	0.004	
**tectum**	to (4,7)	Right	Mesencephalon	1.23	0.191	
**nucleus interpeduncularis**	nip (4)	Right	Mesencephalon	0.004	0.002	
**hypothalamus ventralis**	hyt v (4)	Right	Mesencephalon	0.551	0.067	
**pituitary**	hy (9,10)	Right	Pituitary	0.09	0.018	
**rhombencephalon**	rh (9)	Right	Rhombencephalon	0.516	0.058	
**nervous trigeminus**	V (4)	Right	Rhombencephalon	0.07	0.009	
**nervous lateralis anterior/nervous octavus**	VIII (4)	Right	Rhombencephalon	0.154	0.022	
**gray matter of medulla oblongata**	gmob (9)	Right	Rhombencephalon	0.687	0.076	
**withe matter of medulla oblongata**	wmob (9)	Right	Rhombencephalon	1.349	0.151	

Bibliography:

1. Wang, H. H., Li, L. Y., Wang, L. W. & Liang CC. Morphological and histological studies on the telencephalon of the salamander Onychodactylus fischeri. Neurosci Bull. 23(3):170-174. 2007.

2. Mühlenbrock-Lenter, S., Roth, G. & Laberge F. Evolution of the Pallium in Amphibians. In: Binder M.D., Hirokawa N., Windhorst U. (eds) Encyclopedia of Neuroscience. Springer, Berlin, Heidelberg. (2009).

3. Amamoto, R., et al. Adult axolotls can regenerate original neuronal diversity in response to brain injury. Elife. 2016;5:e13998.

4. Clairambault, P., et al. Organization of the serotoninergic system in the brain of two amphibian species, *Ambystoma mexicanum* (Urodela) and *Typhlonectes compressicauda* (Gymnophiona). Anat Embryol 190, 87–99 (1994).

5. Laberge, F., Mühlenbrock-Lenter, S., Grunwald, W., Roth, G. Evolution of the Amygdala: New Insights from Studies in Amphibians. Brain Behav Evol. 67:177-187. 2006.

6. Krug, L., Wicht, H. & Northcutt, R. G. Afferent and efferent connections of the thalamic eminence in the axolotl, *Ambystoma mexicanum*. Neurosci Lett. 149(2):145-148. 1993.

7.- Beltramo, M., et al. Immunolocalization of aromatic L-amino acid decarboxylase, tyrosine hydroxylase, dopamine, and serotonin in the forebrain of *Ambystoma mexicanum*. J Comp Neurol. 1998;391(2):227-247.

8.- Maden, M., Manwell, L. A. & Ormerod BK.. Proliferation zones in the axolotl brain and regeneration of the telencephalon. Neural Development. Jan 17;8:1. (2013).

9.- Dicke, U., Wallstein, M. & Roth, G. 5-HT-like immunoreactivity in the brains of plethodontid and salamandrid salamanders *(Hydromantes italicus*, *Hydromantes genei*, *Plethodon jordani, Desmognathus ochrophaeus, Pleurodeles waltl*): an immunohistochemical and biocytin double-labelling study. Cell Tissue Res 287, 513–523 (1997).

10.- Bidaud I, Galas L, Bulant M, Jenks BG, Ouwens DT, Jégou S, Ladram A, Roubos EW, Tonon MC, Nicolas P, Vaudry H. Distribution of the mRNAs encoding the thyrotropin-releasing hormone (TRH) precursor and three TRH receptors in the brain and pituitary of *Xenopus laevis*: effect of background color adaptation on TRH and TRH receptor gene expression. J Comp Neurol. 2004 Sep 6;477(1):11-28. doi: 10.1002/cne.20235. PMID: 15281077.
